# Surgery for Young Adults With Aortic Valve Disease not Amenable to Repair

**DOI:** 10.3389/fsurg.2018.00018

**Published:** 2018-03-02

**Authors:** Mustafa Zakkar, Vito Domanico Bruno, Alexandru Ciprian Visan, Stephanie Curtis, Gianni Angelini, Emmanuel Lansac, Serban Stoica

**Affiliations:** ^1^Departments of Cardiology and Cardiothoracic Surgery, Bristol Heart Institute, Bristol Royal Infirmary, Bristol, United Kingdom; ^2^Department of Cardiac Surgery, L'Institut Mutualiste Montsouris, Paris, France

**Keywords:** aortic valve replacement, ross operation, homograft, young adult, aortic stenosis

## Abstract

Aortic valve replacement is the gold standard for the management of patients with severe aortic stenosis or mixed pathology that is not amenable to repair according to currently available guidelines. Such a simplified approach may be suitable for many patients, but it is far from ideal for young adults considering emerging evidence demonstrating that conventional valve replacement in this cohort of patients is associated with inferior long-term survival when compared to the general population. Moreover; the utilisation of mechanical and bioprosthetic valves can significantly impact on quality and is linked to increased rates of morbidities. Other available options such as stentless valve, homografts, valve reconstruction and Ross operation can be an appealing alternative to conventional valve replacement. Young patients should be fully informed about all the options available - shared decision making is now part of modern informed consent. This can be achieved when referring physicians have a better understanding of the short and long term outcomes associated with every intervention, in terms of survival and quality of life. This review presents up to date evidence for available surgical options for young adults with aortic stenosis and mixed disease not amenable to repair.

## Introduction

Current guidelines recommend prosthetic valves replacement as the gold standard for the management of patients with severe AS or mixed pathology while dividing recommendations based on single age cut off (1, 2). Such a simplified approach may be suitable for many patients but it is far from ideal for young adults.

It is recognised that aortic pathology differs between age groups. In fact, younger people have high incidence of bicuspid valves, sometimes associated with additional aortopathy. Furthermore; some young patients have a small annulus which creates its own set of problems. Prosthetic valve replacement may not be the best option for young adults as it can be associated with complications such as thromboembolism, bleeding and limited durability. Moreover; the use of prosthetic valves in such patients can lead to prosthetic-patient mismatch when annular enlargement is not performed which may in severe cases potentially impact adversely on long term outcomes (3, 4). Importantly, most long-term studies of AVR include heterogeneous cohorts which makes their results challenging to extrapolate to young adults specifically.

This review of literature based on published article focuses on the surgical options for young adults (<65 years) with aortic stenosis and/or mixed disease not amenable to repair and discusses stented and stentless valves, valvuloplasty, homografts, the Ross operation and complete valve reconstruction. We aim to provide a balanced over view of available data and discuss important papers of interest.

## Surgical Options

### Stented Tissue or Mechanical Valve Replacement

The use of stented prostheses in young adults undergoing AVR is not without its drawbacks as these valves may have a suboptimal haemodynamic profile alongside the problem of rapid degeneration for tissue prostheses and the need for life long anticoagulation with mechanical valves. Some studies reported that AVR results in normal long-term survival, with low rates of prosthesis-related complications (5, 6). Such studies however included a wide range of ages or did not subdivide patients by age. A closer look at reports in young adults shows a different picture.

**Figure 1:**
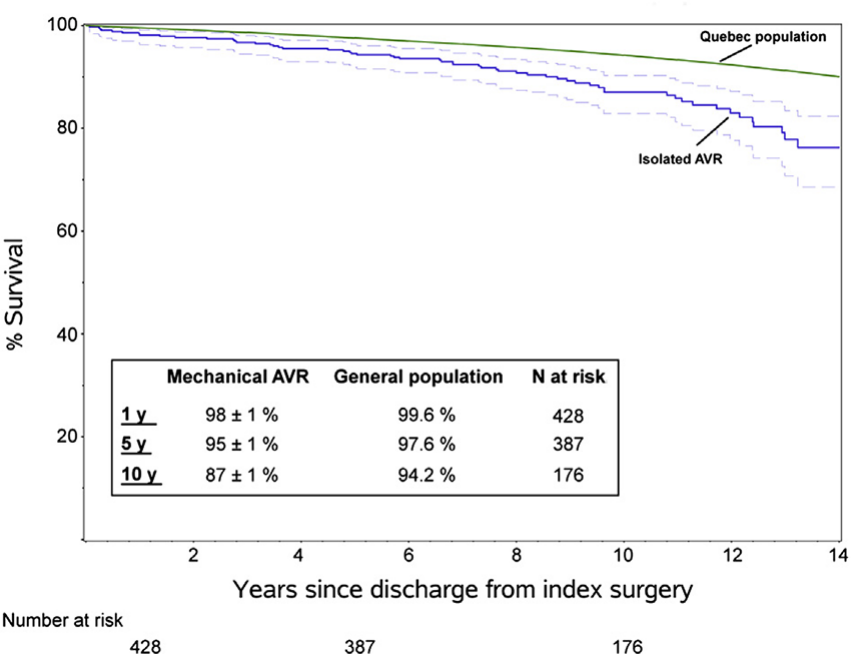
Survival in young adults undergoing isolated mechanical AVR compared to sex and age matched population (Bouhout et al.) (7) (printed with permission from JTCVS).

Bouhout et al. (7) reported 450 consecutive adults <65 years (53 ± 9 years) who underwent elective isolated mechanical AVR, with an overall survival at 1, 5, and 10 years of 98, 95 and 87% respectively, lower than expected for the age- and gender-matched local general population (Figure 1). Actuarial freedom from prosthetic valve dysfunction was 99, 95 and 91% at 1, 5, and 10 years, with freedom from reoperation at 10 years of 82%. Freedom from major haemorrhage was 98, 96 and 90% at 1, 5 and 10 years. It appears that normal life expectancy is not restored in young adults and there is a low but constant hazard of prosthetic valve reintervention.

Ruel et al. (8) examined 500 young adults (18–50 years) who had AVR (*n* = 309) and/or mitral valve replacement (mean age in AVR 39.1 ± 8.1, MVR 41.5 ± 6.7 and combined valve replacement 42.1 ± 6.1). 5, 10 and 15 year survival was 92, 88 and 80% after AVR. The ten-year cumulative incidence of embolic stroke was 6.3% for mechanical AVR vs 6.4% for bioprosthetic AVR patients. Freedom from recurrent heart failure, and freedom from disability were significantly higher in bioprosthetic than mechanical valve patients. Furthermore, career or income limitations, higher prevalence of disability and poorer disease perception were more often linked to a mechanical prosthesis.

With the documented improved durability of contemporary bioprostheses, there has been a massive shift towards using them in younger patients (9). However, long-term outcomes of patients younger than 60 years old are not well known. Forcillo et al. (10) in a series of 144 AVR patients <60 years (51 ± 9), showed actuarial survival rates of 89, 79 and 57% after 5, 10 and 15 years of follow-up, respectively. This too was lower than a gender- and age-matched general population at all time points. At 5, 10 and 15 years freedom from major adverse cardiac events was 89, 87 and 75% whereas freedom from prosthetic valve dysfunction was 97, 84 and 57%. Similarly, Welke et al. (11) reported 2,168 patients who had a Carpentier-Edwards aortic valve looking specifically at the effect of age, which was the independent variable most significantly associated with re-operation. There was an early hazard phase for patients between 21–49 years of age, such that the freedom from re-operation was 89% at 3 years. By 10 years this was only 58% in 21–49 years old, compared with 68% for 50–64 years, 93% for 65–74 years, and 99% for patients >75 years.

The phenomenon of higher mortality after AVR was observed in a national cohort in Sweden by Kvidal et al in 2359 patients (mean age 63.2 years for male and 67.4 years for female) (12) the observed vs expected death ratios being higher in younger patients.

It is important however to point that these studies investigating the impact of stented valve replacement on long-term outcomes almost always contain a wide range of ages, thus extrapolation of strong conclusions to young adults cohort should be taken with cautious as such heterogeneity can impact on results. Additionally the rapid development of new stented valves with superior leaflet preparation and well as dilatable sewing rings may in the future provide an acceptable valve substitute for the younger patients that can improve outcomes (13).

### The Ross Operation

The Ross operation is an appealing underutilised option for valve replacement in young adults (14, 15), perhaps due to concerns about operative risk and the perceived need for reintervention. Although it is technically more complex, it does have excellent short and long-term outcomes.

Skillington et al. (16) presented a large series of 324 adults undergoing a Ross operation of whom 204 patients [mean age of 41.3 years (16–62)] underwent this procedure for either AS or mixed pathology. There was no early mortality and at 15 years results showed 98% survival, 99% freedom from re-operation on the aortic valve and 97% freedom from re-operations on the aortic and pulmonary valves.

**Figure 2:**
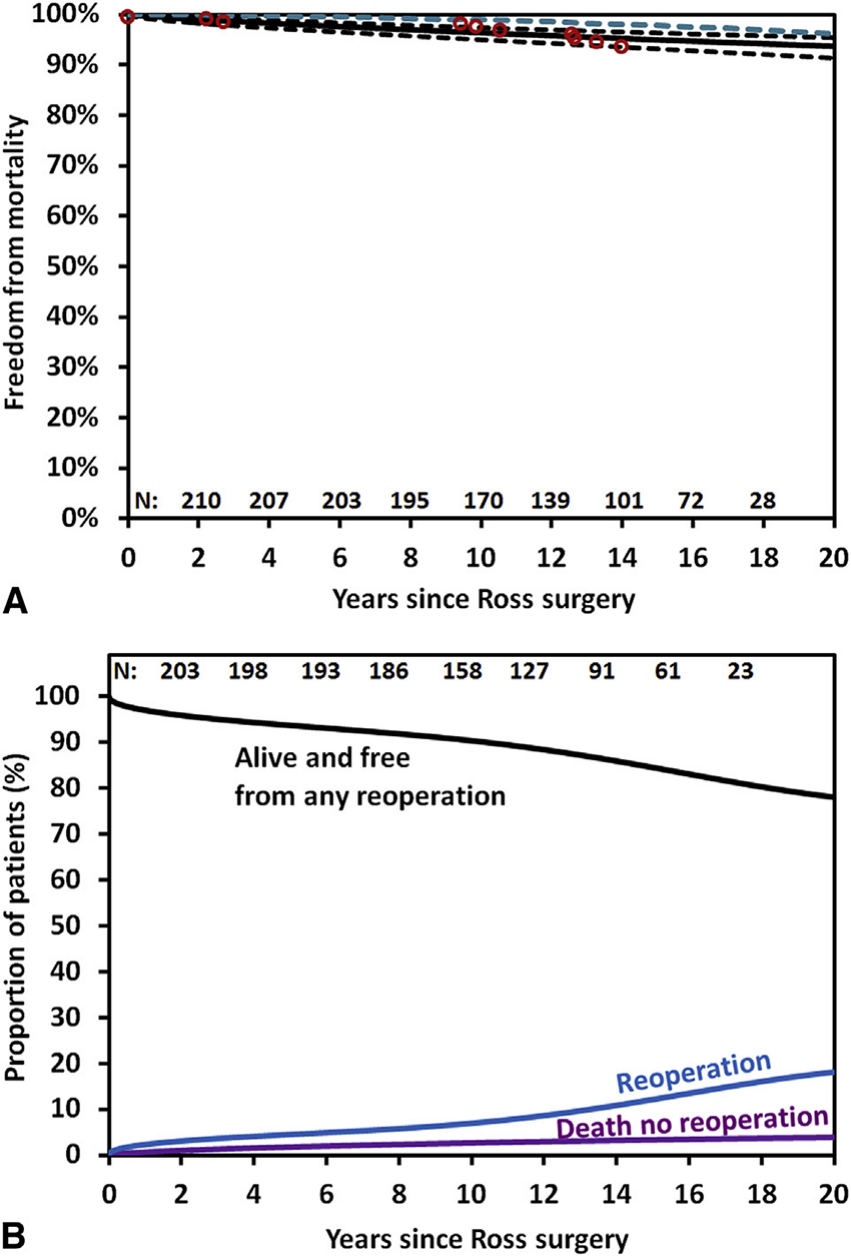
**(A)** Survival estimates of patients who underwent the Ross procedure, including those who no longer had the pulmonary autograft (*black solid line*) with 95% confidence limits (*black dotted line*) and that of the general population matched for age and sex (*dotted blue line*). **(B)** Reoperation-free survival and the competing risks for any reoperation on the pulmonary autograft or homograft and death (David et al.) (pernited with permission from JTCVS) (17).

David et al. (17) demonstrated excellent results in 212 patients who underwent the Ross operation with a mean age of 34 ± 9 years. Survival at 20 years was 93.6%, similar to the matched general population. Freedom from reoperation on the autograft was 81.8% and on the pulmonary homograft was 92.7%, and in both was 79.9% at 20 years (Figure 2A, B). Similar results were obtained from the German Ross registry (18), which included 1,779 adult patients (mean age 44.7 ± 11.6 years) with mean follow up of 8.3 years (range 0–24.3 years). In this study, the early mortality was 1.1% and late survival of the adult population was comparable with the matched general population. Overall freedom from reoperation was 94.9, 91.1 and 82.7% at 5, 10 and 15 years. Freedom from autograft reoperation was 96.8, 94.7 and 86.7% and freedom from homograft reoperation was 97.6, 95.5 and 92.3%, at 5, 10 and 15 years.

The recent analysis of the UK national audit included a 3-way propensity-matched comparison and indicated superiority of the Ross over both mechanical and tissue AVR, bioprostheses being associated with the worst outcomes (19).

These series reinforce previously published results showing low hospital mortality and excellent long-term survival with the Ross operation (17, 19, 20). One of its advantages is the diminished risk of thromboembolism and no need for anticoagulation which impacts positively on quality of life (21, 22). Moreover, it is able to accommodate different aortic pathologies except for severe connective tissue disorders and certain rheumatic patients (23–28).

As for the mechanism underlying these outcomes, it remains speculative. It is possible that there are several factors at play as the differences in survival are hard to explain only by attrition related to anticoagulation in the mechanical valves or structural degeneration in tissue valves. Pibarot (29) elegantly showed how the autograft retains a remarkable ability to eliminate a transvalvular gradient even at peak exercise so its physiological properties are likely to be an important reason. Moreover; this has been recently supported by a met-analysis comparing valve haemodynamics in conventional AVR compared to Ross (30).

Reintervention remains the main concern as there is a perception that the rates are high and this may be associated with increased morbidity and mortality (14, 15). Reports on outcomes of reintervention after Ross however persistently demonstrated that it can be performed safely and that the autograft itself can sometimes be salvaged by repair or even valve-sparing reimplantation (31–33).

There are several methods for implanting the autograft, including subcoronary insertion, the inclusion technique and the free-standing root (with its own variations, but overall the most common variant). All of these provide comparable outcomes and the choice seems to be operator-dependent to an extent (16, 34). It should be noted that the procedure can be complemented by Kono annular enlargement which deals effectively with a small annulus or left ventricular outflow tract (35, 36). It is perhaps conceivable that some conventional AVRs end up with potentially severe patient-prosthetic mismatch with adverse consequences for function and survival, especially in younger patients (3, 4, 37). In terms of the pulmonary conduit, there is no complications-free option which is one of the major draw-backs of Ross operation considering that it potentially creates double valve disease specially in the pulmonary position which is usually replaced with pulmonary homograft treated or freshly decellularised homografts. In fact, when available, the use of freshly decellularised pulmonary homografts can be associated with improved freedom from reoperation as well as exhibiting adaptive growth both at early and mid-term (38, 39) which is pivotal when considering the correct conduit to use in young adults. The limited availability of pulmonary homografts resulted in using different prosthetic, or composite conduits for RV-PA reconstruction, which perform generally well and do not impact negatively on outcomes (40–42).

Although the Ross operation has good results in the right subset of patients, it is important to point that it still can be associated with considerable early and late mortality as been demonstrated by Etnel et al. (43) in a recent meta-analysis reporting outcome after paediatric aortic valve replacement (mean age 9.4 years for Ross) which showed that the Ross operation still associated with suboptimal early and late outcomes. Furthermore; the Ross procedure was also associated with a substantial reoperation rate in the first postoperative decade, and a further increase in reoperation rates is to be expected in the second postoperative decade. Interestingly, it seems that for neonates and infants undergoing the Ross procedure, aortic valve reoperation rates seem to be lower, whereas RV related reoperation rates are 2 times higher compared with older children after the Ross procedure.

### Homografts and Stentless Valves

The use of a homograft as a valve substitute in young adults requiring AVR has multiple advantages, including excellent haemodynamics and good resistance to infection, without the need for anticoagulation. However, there are problems such as the lack of capacity for growth of the valve, the limited supply and functional deterioration requiring reintervention.

There is limited data in young adults and most available series included a mixed age group or a variety of indications. The use of homografts is associated with slightly higher in-hospital mortality rates (2.5–7%) (44–46). Thromboembolic events occur very rarely with excellent freedom from late infection (44–46). Structural deterioration requiring reintervention seems to be the major issue complicating its use (44–46).

The deterioration of homografts, alongside their limited availability, resulted in the utilisation of stentless aortic xenografts as an alternative. Stentless prostheses are technically demanding too but allow improvements in transvalvular gradients and regression of left ventricular hypertrophy (47, 48). Another advantage is their off-the-shelf availability.

There is limited evidence on stentless valves in young adults, most studies reporting mixed ages and/or pathologies. Christ et al. (49) reported 188 stentless AVR patients with a mean age of 53.1 ± 7.1 years and 63.3% stenosis or mixed lesions. Hospital mortality was 2.5% for isolated AVR and actuarial survival and freedom from reoperation at 10 years were 70 and 83% respectively. In another study the same group from Toronto showed excellent haemodynamic results without significant rise of transvalvular pressure gradients or significant regurgitation until 14 years after implantation, and sustained improvements in left ventricular mass and function (50).

Bach et al. (51) in a study of the outcomes of stentless valves in different age ranges, demonstrated excellent survival for patients aged ≤60 years compared to those aged ≥61 years. Freedom from cardiac death was 94.6 and 70.7%, respectively. Freedom from structural valve deterioration was similar between the two age groups (<90%) and there was no significant difference in freedom from reoperation at 12 years between younger and older patients.

### Aortic Valvulotomy and Valvuloplasty

Valvulotomy can be done surgically or percutaneously. Open valvulotomy is not common practice in the current era for young adults and was previously considered a palliative treatment, with reoperation needed in 25–40% of patients with in 10 years (52–54). Balloon valvuloplasty can be a safe and effective treatment for children presenting with congenital AS. It confers palliative benefits by providing a reduction in gradient ranging from 49–70%, thus delaying the time for more definite surgical intervention (53, 54). Currently this is less appealing compared to other interventions as there is a significant risk of developing severe aortic incompetence and there is a need for repeat balloon valvulotomy or definitive intervention. Percutaneous procedures are used commonly in children but even in this subgroup there has been a resurgence of open valvuloplasty, even in neonates (54–56).

### Complete Valve Reconstruction

Complete replacement of aortic leaflets was described decades ago (57, 58). Initial attempts to use autologous pericardium were associated with retraction and fibrosis (57). The technique was abandoned until introduction of glutaraldehyde treatment, which has a strengthening effect (58). Al Halees et al (59) reported 65 young patients [mean age 33 years (12–68)] out of 92 patients who underwent valve construction with such autologous pericardial reconstructions; the hospital mortality was 2% and at 10 years freedom from reoperation was 72% with freedom from structural valve degeneration of 80%.

More recent derivations such as the Ozaki operation may potentially play a role in treating young adults. In a series of total aortic valve leaflets creation in the young adults subgroup (mean age 47.8 ± 11.2), there were no in-hospital deaths or thromboembolic events, with freedom from reoperation of 98.9% at 76 months of follow-up (60).There is increased interest in the Ozaki technique as the use of dedicated sizers makes it reproducible plus there is hope related to new biomaterials. The use of decellularised matrix for valve cusps reconstruction can be an appealing option especially as the technology is evolving to aid guided tissue regeneration by means of autologous reseeding. The combining of valve reconstruction techniques such as Ozaki with decellularised pericardial patch can be an interesting development that can provide good alternative to conventional valve replacement in young adult as at least it seems it can provide adequate haemodynamics with sustained mechanical integrity and limited calcifications in animal models (61).

## Comparing Available Surgical Options

Some direct comparisons have been made between various surgical options. Most are retrospective studies in well matched cohorts but some randomised studies have been carried out.

### Homografts and Tissue Valves

In young adults it appears that stentless valves may have similar clinical performance to homografts during follow up (62, 63). The differences between stented and stentless valves are less clear. There is some evidence demonstrating no significant differences in haemodynamic function or clinical events between the two types of valves (64, 65), whereas other studies showed better outcomes with stentless prostheses (66–68).

### Conventional AVR vs Ross

There are no randomised studies comparing outcomes of conventional AVR to the Ross operation. Propensity-matched cohorts showed no differences in mortality or major adverse perioperative outcomes in Ross vs mechanical AVR but long-term outcomes are less clear (69, 70). Additionally in the Toronto series the Ross procedure was better than propensity-matched mechanical AVR in terms of freedom from cardiac- and valve-related mortality (69). Even though long-term survival and freedom from reintervention appear comparable between Ross and mechanical AVR, the quality of life after Ross has been shown to be superior (21). The Ross procedure is associated with improved freedom from cardiac and valve-related mortality and a significant reduction in the incidence of stroke and major bleeding (17, 19, 71). These are major adverse outcomes that should be taken into consideration when considering what the best option is for a young adult with decades of life, and thus risk, ahead.

### Ross vs Homografts

**Figure 3:**
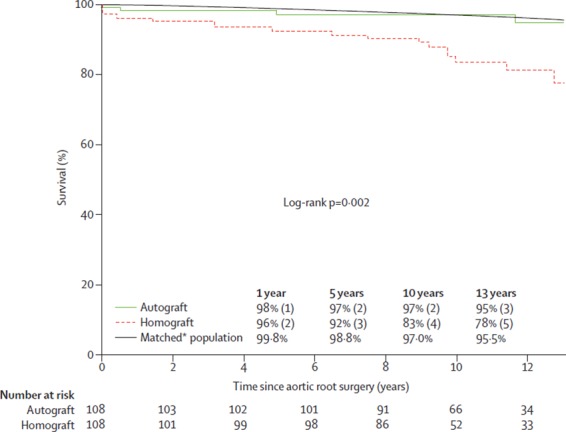
Actuarial survival after autograft versus homograft aortic root replacement (El-Hamamsy et al.) (72) (printed with permission from Lancet).

To date there is only one single centre randomised trial of the Ross operation and the comparator procedure was another biological root solution (homograft): 216 patients, mean age 38.5 years (72). There was no difference in perioperative mortality between Ross and homograft (<1 vs 3%, *p* = 0.621). However, actuarial survival was superior in the Ross group at 10 years (97 vs 83%) and similar to an age and sex-matched population. Moreover, in multivariate analysis, the only independent predictor of late mortality was homograft use (Figure 3). This important result from a randomised comparison is indicative that selection bias cannot be solely responsible for better results in Ross cohorts.

## Conclusion

Young adults requiring aortic valve intervention represent an increasing problem. Guidelines recommend prosthetic replacement with either mechanical or tissue valves. This approach is too simplistic and is based on studies that were heterogeneous and did not consider how the expected long life span for such patients can be adversely affected. New evidence confirms that conventional AVR in young adults is associated with inferior long-term survival when compared to the general population. The main options of mechanical and tissue valves are also limited by complications related in the main to anticoagulation and accelerated structural degeneration respectively. Other options such as stentless valve and homografts can be considered but there is no supportive evidence for their superiority to the Ross operation, data being generally scarce in young patients. In practice homografts are often reserved for patients with endocarditis, which may partly explain the higher early mortality (Table 1).

**Table 1:** Summary of papers discussed in the review

	**Number of patients**	**Age (years)**	**Intervention**	**Main outcome**
**Bouhout et al. (7)**	450	53 ± 9	Mechanical AVR	Normal life expectancy is not restored in young adults after AVR. There is a low but constant hazard of prosthetic valve reintervention
**Ruel et al. (8)**	309 AVR group.40 Double valve group.	39.1 ± 8.1 years for AVR group42.1 ± 6.1 years for double valve group	Stented valve replacement in aortic and/or mitral position	Late outcomes of modern prosthetic valves in young adults remain suboptimal
**Forcillo et al. (10)**	144	50 ± 9	AVR with bioprosthesis	Late survival was inferior to an age- and gender-matched population.Structural valve deterioration and the need for reintervention were common late after implantation
**Welke et al. (11)**	2,168	Compared different age21–4950–6465–74>75	AVR with bioprosthesis	There was an early hazard phase for patients between 21–49 years of age, such that the freedom from re-operation was 89% at 3 years. By 10 years freedom from intervention 58% in 21–49 years compared with 68% for 50–64 years, 93% for65–74 years.
**Kvidal et al. (12)**	2,359	63.2 for male and 67.4 for female	stented valve replacement	The observed vs expected death ratios are higher in younger patients
**Skillington et al. (16)**	324	41.3 (16–62)	Ross operation	Ross operation results in excellent freedom from re-operation on the aortic valve at 15 years
**David et al. (17)**	212	34 ± 9	Ross operation	Survival after the Ross procedure is similar to the general population.Pulmonary homograft dysfunction is common at 20 years
**Sievers et al. (18)**	1,779	44.7 ± 11.6	Ross operation	Long-term survival is comparable with that of the age- and gender-matched general population.Reoperation rates are within the 1%/patient-yearBoundaries
**Notzold et al. (21)**	40 in Ross group.40 in mechanical AVR group	57.6 ± 10.3 ross group.59.2 ± 10.4 mechanical AVR group	Ross compared to mechanical AVR	Ross is associated with better quality of life
**Etnel et al. (43)**	2,409 Ross group.696 mechanical AVR group.224 homograft group.	9.4 in Ross12.8 in mechanical AVR.8.9 in homograft group.	Meta-analysis of different techniques	Available aortic valve substitutes are associated with suboptimal results in children
**Christ et al. (49)**	188	53.1 ± 7.1	Stentless valve	Hospital mortality 2.5% for isolated AVR.Actuarial survival and freedom from reoperation at 10 years are 70 and 83% respectively
**Bach et al. (51)**	Total 725	≤60 in 57 patients.	Stentless valve	Excellent survival for patients aged ≤60 years.Freedom from structural valve deterioration was similar between the two age groups (<90%) and there was no significant difference in freedom from reoperation at 12 years between younger and older patients. compared to those aged ≥61 years
**AL Halees et al. (59)**	92 underwent reconstruction. 65 had treated autologous pericardium.27 treated bovine pericardium.	33.3 (12–68)	Valve reconstruction	Aortic valve reconstruction is feasible with good haemodynamics, low mortality and thromboembolic rate.Function at 10 years is comparable to stentless bioprosthesis
**Ozaki et al. (60)**	108	47.8 ± 11.2	Valve reconstruction	Excellent outcomes after total valve reconstruction
**Bouhout et al. (69)**	70 in Ross group.70 in mechanical AVR group.	Matched age was 52 ± 14 M, 52 ± 13 Ross	Propensity matching mechanical AVR to Ross	No difference in mortality or major outcomes
**El-Hamamsy et al. (72)**	216	39 (19–68) in homograft group.38 (19–66) in autograft group	Ross vs Homograft	Autografts can significantly improve long term outcomes in patients

The multitude of options is partly indicative of the lack of strong evidence to support one option over the others, the most reliable literature being based on matched designs with very few randomised trials. There is no ideal valve substitute and it is difficult to provide general recommendations in this issue which can explain the simplistic approach of the current guidelines. However, there is evidence to support the utilisation of the Ross operation in selected subset of patients and maybe it can be something that should be considered on individual basis, as it does not appear to have any trade-off between survival and quality of life but it remains an operation that can create two valve pathology. Concerns regarding the high incidence of reintervention after Ross are not supported with strong evidence; reintervention, when needed, can be done safely.

A multi-centre randomised control trial to compare outcomes of the Ross procedure with prosthetic valves is needed in this current era, as more bioprosthetic valves are implanted in younger patients and the role to transcatheter implants is expanding to include intermediate risk or even young and low risk patients in the future (22, 73). Finally, young patients should be fully informed about all the options available - shared decision making is now part of modern informed consent. This can be achieved when referring physicians have a better understanding of the short and long-term outcomes associated with every intervention, in terms of both quantity and quality of life (74).

## Author Contributions

MZ planned, prepared and wrote the manuscript. VB wrote the draft. AV conducted research of past papers and prepared the draft. SC reviewed the draft and rewrote the article. GA reviewed the draft and also contributed to the rewrite. EL reviewed the draft and contributed to the rewrite. SS planned, prepared and wrote the manuscript.

## Conflict of Interest Statement

The authors declare that the research was conducted in the absence of any commercial or financial relationships that could be construed as a potential conflict of interest.

## Abbreviations

AVR: Aortic valve replacement
AS: Aortic stenosis
